# Mutation signature analysis identifies increased mutation caused by tobacco smoke associated DNA adducts in larynx squamous cell carcinoma compared with oral cavity and oropharynx

**DOI:** 10.1038/s41598-019-55352-y

**Published:** 2019-12-17

**Authors:** Andrew P. South, Nicoline Y. den Breems, Tony Richa, Uche Nwagu, Tingting Zhan, Shiv Poojan, Ubaldo Martinez-Outschoorn, Jennifer M. Johnson, Adam J. Luginbuhl, Joseph M. Curry

**Affiliations:** 1 Department of Dermatology and Cutaneous Biology, Thomas Jefferson University, Philadelphia, PA 19107 USA; 2 The Joan and Joel Rosenbloom Research Center for Fibrotic Diseases, Thomas Jefferson University, Philadelphia, Pennsylvania, PA 19107 USA; 3 Sidney Kimmel Cancer Center, Thomas Jefferson University, Philadelphia, PA 19107 USA; 4 Center for Advanced Computing (C-fACS), Lincoln University, Lincoln, 7647 New Zealand; 5 Department of Otolaryngology – Head and Neck Surgery, Thomas Jefferson University, Philadelphia, PA 19107 USA; 6 Department of Pharmacology and Experimental Therapeutics, Thomas Jefferson University, Philadelphia, PA 19107 USA; 7 Department of Oncology, Thomas Jefferson University Philadelphia, Philadelphia, PA 19107 USA

**Keywords:** Cancer genetics, Cancer genetics

## Abstract

Squamous cell carcinomas of the head and neck (HNSCC) arise from mucosal keratinocytes of the upper aero-digestive tract. Despite a common cell of origin and similar driver-gene mutations which divert cell fate from differentiation to proliferation, HNSCC are considered a heterogeneous group of tumors categorized by site of origin within the aero-digestive mucosa, and the presence or absence of HPV infection. Tobacco use is a major driver of carcinogenesis in HNSCC and is a poor prognosticator that has previously been associated with poor immune cell infiltration and higher mutation numbers. Here, we study patterns of mutations in HNSCC that are derived from the specific nucleotide changes and their surrounding nucleotide context (also known as mutation signatures). We identify that mutations linked to DNA adducts associated with tobacco smoke exposure are predominantly found in the larynx. Presence of this class of mutation, termed COSMIC signature 4, is responsible for the increased burden of mutation in this anatomical sub-site. In addition, we show that another mutation pattern, COSMIC signature 5, is positively associated with age in HNSCC from non-smokers and that larynx SCC from non-smokers have a greater number of signature 5 mutations compared with other HNSCC sub-sites. Immunohistochemistry demonstrates a significantly lower Ki-67 proliferation index in size matched larynx SCC compared with oral cavity SCC and oropharynx SCC. Collectively, these observations support a model where larynx SCC are characterized by slower growth and increased susceptibility to mutations from tobacco carcinogen DNA adducts.

## Introduction

Squamous cell carcinomas (SCC) arise in tissues that form a barrier between an organism and its environment such as the oral cavity, esophagus, upper airways of the lung, cervix, vulva, urethra and skin^1^. In 2017, excluding skin SCC for which figures are not considered, 8.8% of all new cancer cases and 11% of all cancer deaths in the US can be attributed to SCC^2,3^. With the noted exceptions of HPV vaccination, immune checkpoint inhibition and EGFR inhibitors, there are very limited targeted prevention or treatment strategies beyond smoking or alcohol use cessation. Therefore, a better understanding of SCC initiation and progression is needed in order to develop effective prevention, early detection, and treatment strategies.

More than 90% of mucosal tumors of the head and neck are SCC (HNSCC) and arise at distinct anatomical sites such as the oral cavity, oropharynx, larynx and hypopharynx. Half of all primary HNSCC tumors will recur and 5 year survival is around 50–60% leading to substantive mortality figures worldwide^4,5^. However, overall survival figures differ by major anatomical sub-site and a number of studies demonstrate larynx SCC have overall improved 5 year survival compared with oral cavity tumors^6,7^, suggesting that SCC arising at different locations within the upper aero-digestive tract are distinct entities with respect to incidence and outcome.

HPV positive HNSCC are prevalent in the oropharynx, where a large proportion of tonsil and base of tongue SCC are virally driven. It is recognized that HPV infection represents a subset of HNSCC with distinct presentation, etiology and outcome^5,8^. Indeed, comparing all SCC from diverse anatomical sites identifies HPV positive tumors (principally from the head and neck and cervix) as a clear genetic subclass of SCC^1^. However, recent data does suggest that HPV infection in the larynx, hypopharynx, or oral cavity, does not change overall prognosis^9,10^, while in the oropharynx HPV has prognostic significance^8^, again supporting the idea that anatomical sub-site influences outcomes. For HPV negative tumors further sub-classification can be based on copy number alterations (CNA) or presence of specific mutations and epigenetic variation^11–14^. Often these differences are not exclusive to anatomical location where, for example, identification of *NSD1* and *NSD2* mutations define a sub-class of both oral cavity and larynx SCC^11,12^.

In addition to HPV, tobacco use is a primary risk factor for HNSCC and smoking status at diagnosis is associated with treatment response, risk of recurrence, and survival^7,15,16^. Smoking during treatment can also influence response^17^, and progression free survival decreases as a direct result of tobacco exposure at diagnosis and during therapy^18^. One prominent mechanism of carcinogenesis associated with tobacco exposure is the formation of DNA adducts which are compounds produced when chemicals react with DNA. Normal cellular repair processes remove adducts and DNA is faithfully replicated when a cell divides. However, if repair processes are overwhelmed or are deficient, the DNA adducts can persist and cause mutations during DNA replication^19^.

Signatures of mutation in cancer, based on collective analysis of large numbers of nucleotide changes and their context, can identify the underlying cause of a given cancer or group of cancers^20,21^. In lung carcinomas and HNSCC, tobacco smoke yields a distinct mutation signature dominated by C > A transversion^22^. The 96 nucleotide context of tobacco smoke associated mutations, termed COSMIC signature 4, has been validated experimentally using murine *tp53* mutant fibroblasts exposed to benzo[*a*]pyrene, a prominent tobacco carcinogen^21,22^. These data demonstrate COSMIC signature 4 is indicative of DNA adduct formation and subsequent mutation. Other signatures of mutation identified in HNSCC include those associated with age and natural variation in the genome^23–25^, COSMIC signatures 1 and 5, as well as endogenous deaminases of the AID (activation-induced cytidine deaminase) and APOBEC (apolipoprotein B mRNA editing enzyme, catalytic polypeptide) family^20,26,27^. Because of their association with age and somatic mutation during the lifespan of an individual, signatures 1 and 5 have been referred to as “clock-like” which could theoretically be used to predict the age of a tissue or tumor^23–25^. Indeed, serial passaging of cells in culture readily induces mutations associated with signature 5^28^. Here we use mutation signature analysis to highlight distinct differences between major anatomical subsets of head and neck cancer and identify larynx SCC as a separate entity with regard to proliferation and mutation susceptibility.

## Results

### Tobacco mutation signatures associated with DNA adduct formation are significantly enhanced in laryngeal SCC compared with all other head and neck SCC

**Figure 1:**
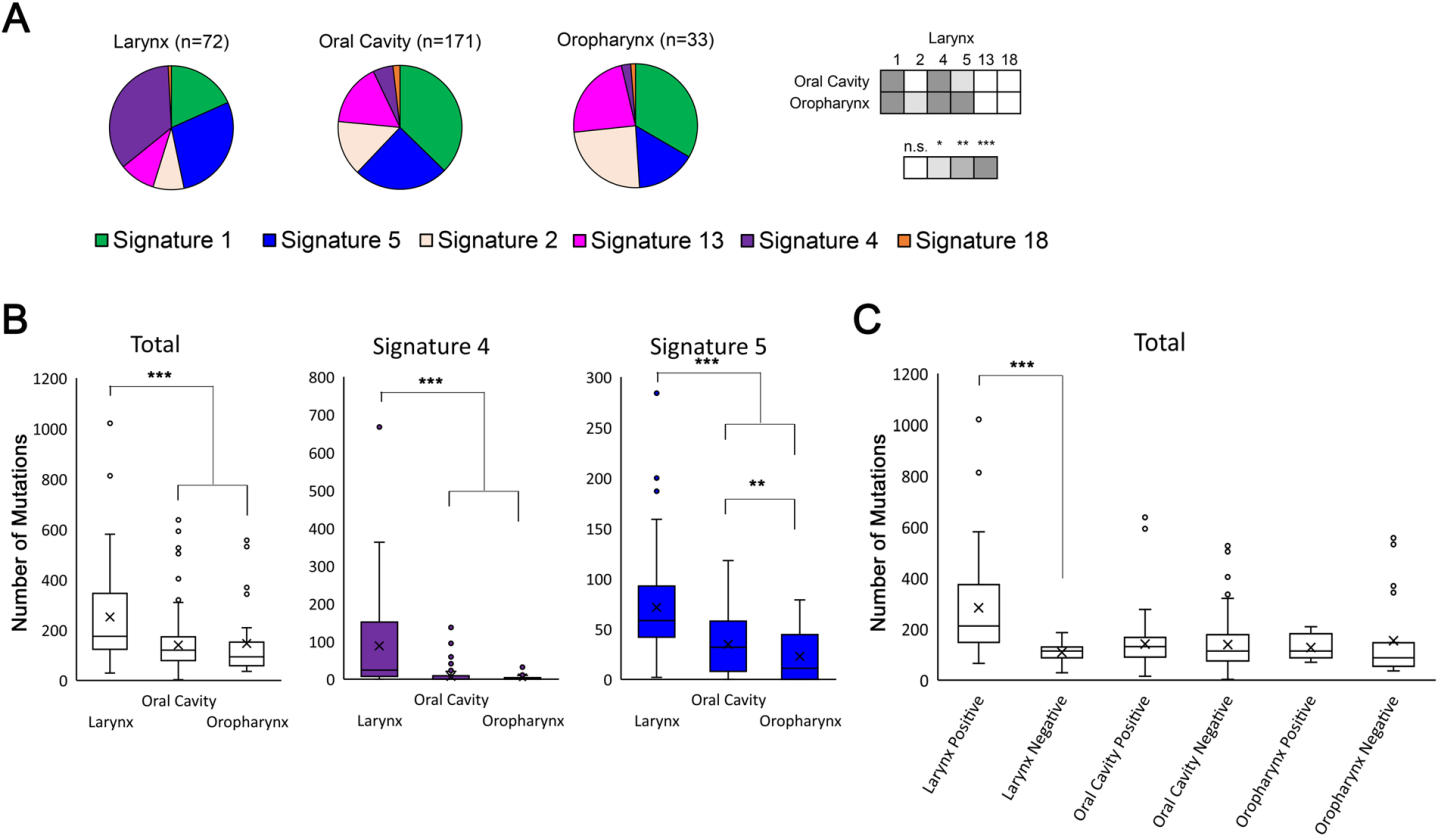
COSMIC signature 4 contributes to significantly higher mutation burden in larynx compared with oral cavity and oropharynx SCC. (**A**) Pie charts show the proportion of all single nucleotide mutations attributed to each of the six COSMIC mutation signatures identified in head and neck SCC for each of the three major sub-sites. n = total number of individual tumors for each sub-site. Signatures 1, 2, 4, 5, 13, and 18, are derived from version 2 of COSMIC mutational signatures. Signatures 1 and 5 are of unknown etiology and associated with age, signature 2 and 13 are associated with APOBEC mutagenesis, signature 4 is associated with tobacco smoke exposure, and signature 18 is associated reactive oxygen species. Matrix to the right of pie charts shows statistical significance of individual COSMIC signature weight (normalized to mutation number) comparing larynx with oral cavity and oropharynx tumors. No significance was seen comparing signature weight between oral cavity and oropharynx tumors for any of the six signatures. n.s. = no significance. (**B**) Box and whisker graphs show total number of single nucleotide mutations as well as those attributed to signature 4 and signature 5, identified in each of the major sub-sites of HNSCC. (**C**) Total mutations stratified by the presence (positive) or absence (negative) of signature 4 mutations. x = mean. *p < 0.05, **p < 0.01, ***p < 0.001.

In our recent examination of mutation signatures in HNSCC arising in non-sun exposed sites, we observed striking sub-site specificity to the presence of tobacco smoke-associated mutations (COSMIC signature 4, the signature associated with tobacco exposure^27^, Fig. 1A and Supplementary Fig. S1A). Larynx SCC have a significantly greater proportion of COSMIC signatures 4 and 5 compared with oral cavity and oropharynx SCC, as well as a concomitant significant reduction in signature 1 compared with oral cavity and oropharynx SCC, and a significant reduction in COSMIC signature 2 compared with oropharynx (Fig. 1A). No difference was seen in signature weight comparing oral cavity and oropharynx SCC. Overall, 53% of 278 TCGA (The Cancer Genome Atlas) HNSCC samples^14^ were positive for signature 4, compared with 81% of patients who reported smoking, 63% of whom were current or recently reformed smokers (within 15 years). When anatomical site is considered it can be seen that signature 4 mutations were greatly enriched in the larynx (Fig. 1B) for which 82% of larynx SCC (59/72 tumors) were positive, while only 44% of oral cavity or oropharynx SCC were positive (90/204 tumors). The total number of mutations was also significantly greater in larynx compared with the major anatomical sub-site classification for oral cavity and oropharynx, however, this number was greatly influenced by those larynx tumors which were positive for tobacco-associated mutations (signature 4, Fig. 1B,C and Supplementary Fig. S1B). When HPV status is considered there is a statistical difference between HPV positive and HPV negative HNSCC for total mutations, signature 4 and signature 5 mutations (Supplementary Fig. S2A), however this difference was dependent on larynx tumors where only 1 from 72 is HPV positive (Supplementary Fig. S2B,C). The number of tobacco-associated signature 4 mutations per tumor for those tumors that were signature 4 positive (>0 mutations attributed to signature 4) was significantly higher in larynx (mean 108 signature 4 mutations, n = 59) compared with other sub-sites (mean 15 signature 4 mutations, n = 90; oral cavity mean of 15.5 signature 4 mutations, oropharynx mean of 13.1 signature 4 mutations), or HPV positive tumors (mean 9.4 signature 4 mutations, n = 12) (Fig. 1B and Supplementary Fig. S2). Overall, mutation burden in larynx was greater than other sub-classes of HNSCC and this was dependent on those larynx samples with signature 4 mutations (Fig. 1 and Supplementary Fig. S2). Complete signature assignation per sample is presented as both total numbers and percentage/weight in Supplementary Fig. S3.

### Larynx SCC are dominated by smokers or recently reformed smokers compared with other sub-sites of head and neck SCC

**Figure 2:**
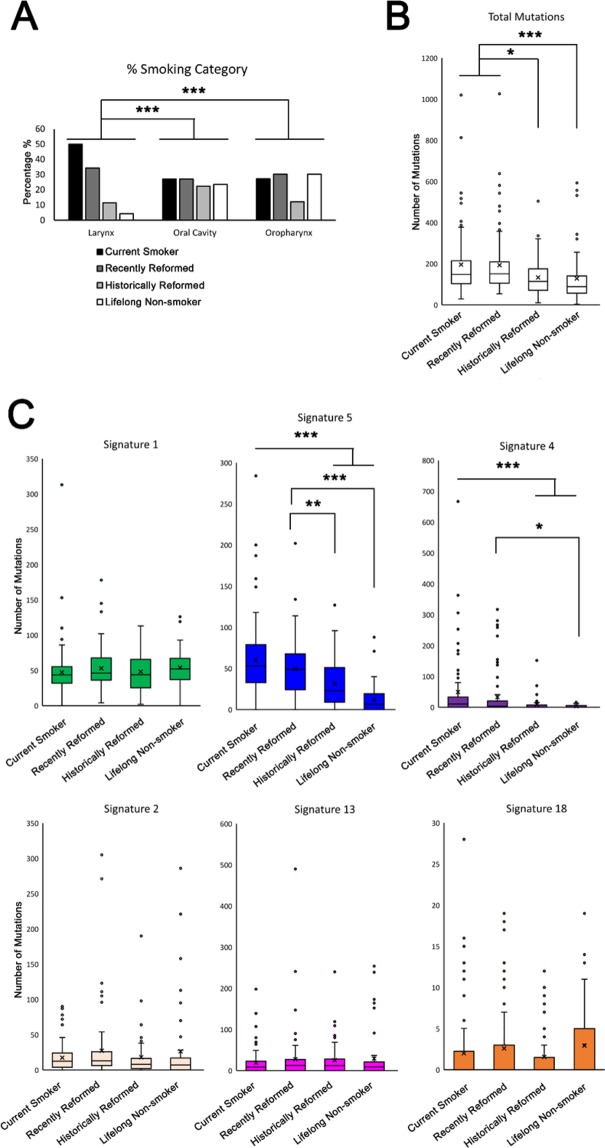
Larynx SCC patients are dominated by smokers or recently reformed smokers and SCC from smokers have significantly more signature 4 and signature 5 mutations compared with non-smokers. Percentage of patients stratified by major anatomical sub-site who are either current smokers, recently reformed smokers (within 15 years), historically reformed smokers (for more than 15 years), or never smokers (**A**). Box and whisker plots show total mutations (**B**) and mutations attributed to individual signatures in HNSCC stratified by smoking status (**C**). Signatures 1, 2, 4, 5, 13, and 18, are derived from version 2 of COSMIC mutational signatures. Signatures 1 and 5 are of unknown etiology and associated with age, signature 2 and 13 are associated with APOBEC mutagenesis, signature 4 is associated with tobacco smoke exposure, and signature 18 is associated reactive oxygen species. x = mean. *p < 0.05, **p < 0.01, ***p < 0.001.

We next compared the incidence of smoking within the TCGA HNSCC data set and between the three major sub-sites of HNSCC: larynx, oral cavity and oropharynx (only 2 hypopharynx samples are included and so were not analyzed). As might be expected, larynx SCC, where more signature 4 mutations are present, had a greater proportion of current smokers or recently reformed smokers compared with oral cavity and oropharynx (p < 0.001, Fig. 2A). Over 80% of larynx samples were from current and recently reformed smokers while this figure was 53% and 58% for oral cavity and oropharynx. Interestingly, while there were more mutations in current and recently reformed smokers across all HNSCC, the overall difference between mutation numbers was not as prominent when comparing sub-site mutation number differences, where larynx stands out as having many more overall mutations (Fig. 2B *cf*. Fig. 1B). The increase in smokers in the larynx SCC cohort might explain the increase in number of signature 4 positive tumors in larynx SCC compared with all other sub-sites but does not account for the increased number of signature 4 mutations per tumor. Comparison of pack years smoked by current and recently reformed smokers showed more pack years in larynx (mean 62 *cf.* 51 in all other HNSCC) but this was not statistically significant.

### Signature 5 and signature 4 mutations correlate with smoking status

In line with previous analysis^22^, only mutations associated with signature 4 (tobacco) and signature 5 (of unknown etiology and previously associated with age in a number of different cancers) showed a relationship with smoking history; current smokers and recently reformed smokers have greater numbers of signature 4 and signature 5 mutations (Fig. 2C). Signature 5 mutation numbers also showed a stepwise reduction from historically reformed smokers (>15 years) and life-long never smokers suggesting a more direct relationship between signature 5 and smoking in HNSCC compared with signature 4 (Fig. 2C).

### Signature 5 correlates with age in HNSCC non-smokers

**Figure 3:**
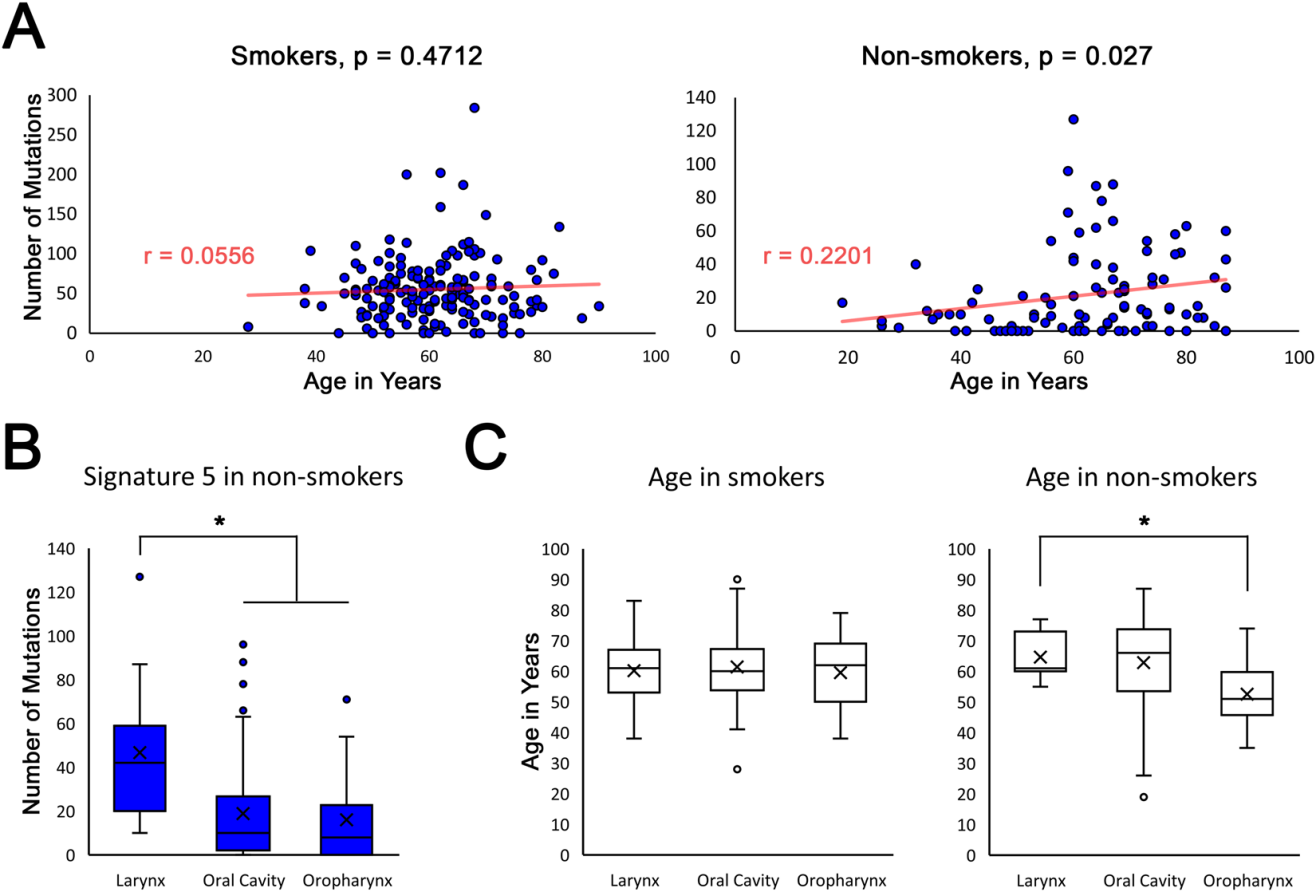
Signature 5 mutations shows positive correlation with age in non-smokers and are enriched in larynx SCC non-smoking patients who are older than non-smoking patients with oropharynx SCC. (**A**) Graphs shows the number of signature 5 mutations (y-axis) plotted against the age (x-axis) of all smokers (left graph, n = 170) or non-smokers (right graph, n = 101) with HNSCC. Pearson Correlation r and p values given. (**B**) Box and Whisker plots show the number of signature 5 mutations identified in HNSCC of non-smokers stratified by major anatomical sub-site. (**C**) Box and Whisker plots showing age of patients with HNSCC stratified by major anatomical sub-site in smokers (left graph) and non-smokers) right graph). Signature 5 is derived from version 2 of COSMIC mutational signatures, is of unknown etiology, and associated with age in certain cancers and somatic tissues. x = mean *p < 0.05.

Previous analysis has demonstrated an association with age and the number of signature 1 mutations in all HNSCC^23^ (Supplementary Fig. S4). We recently showed that tissue-damage associated SCC arising in the skin of patients with the rare blistering disease, recessive dystrophic epidermolysis bullosa (RDEB), show remarkable similarity to HNSCC at the level of mutation signature and transcriptomic analysis, and also show a correlation with age and mutation signature 5 numbers^27^. Given the similarities between RDEB SCC and HNSCC as well as the direct relationship with smoking and signature 5 mutation numbers in HNSCC, we surmised that current or recently reformed smoking status obscures any relationship with age and signature 5 in HNSCC. Indeed, when only lifelong non-smokers or historically reformed smokers are analyzed it can be seen that a significant, positive correlation exists between signature 5 and age (Fig. 3A).

### Larynx SCC non-smokers have significantly greater signature 5 mutations and are generally older compared with other HNSCC non-smokers

Comparing the number of signature 5 mutations in non-smokers (defined as lifelong non-smokers or historically reformed, >15 years), larynx had significantly more signature 5 mutations than either oral cavity or oropharynx (Fig. 3B). In contrast to smokers, non-smoking patients with larynx SCC (n = 11) were significantly older than non-smoking patients with oropharyngeal SCC (n = 14), while non-smoking patients with oral cavity SCC were approximately intermediate in age (n = 76) (Fig. 3C). No difference was seen with signature 1 mutations and major anatomical sub-site in non-smokers (data not shown).

### Laryngeal SCC have significantly less Ki67 positive nuclei than oral cavity or oropharyngeal SCC

**Figure 4:**
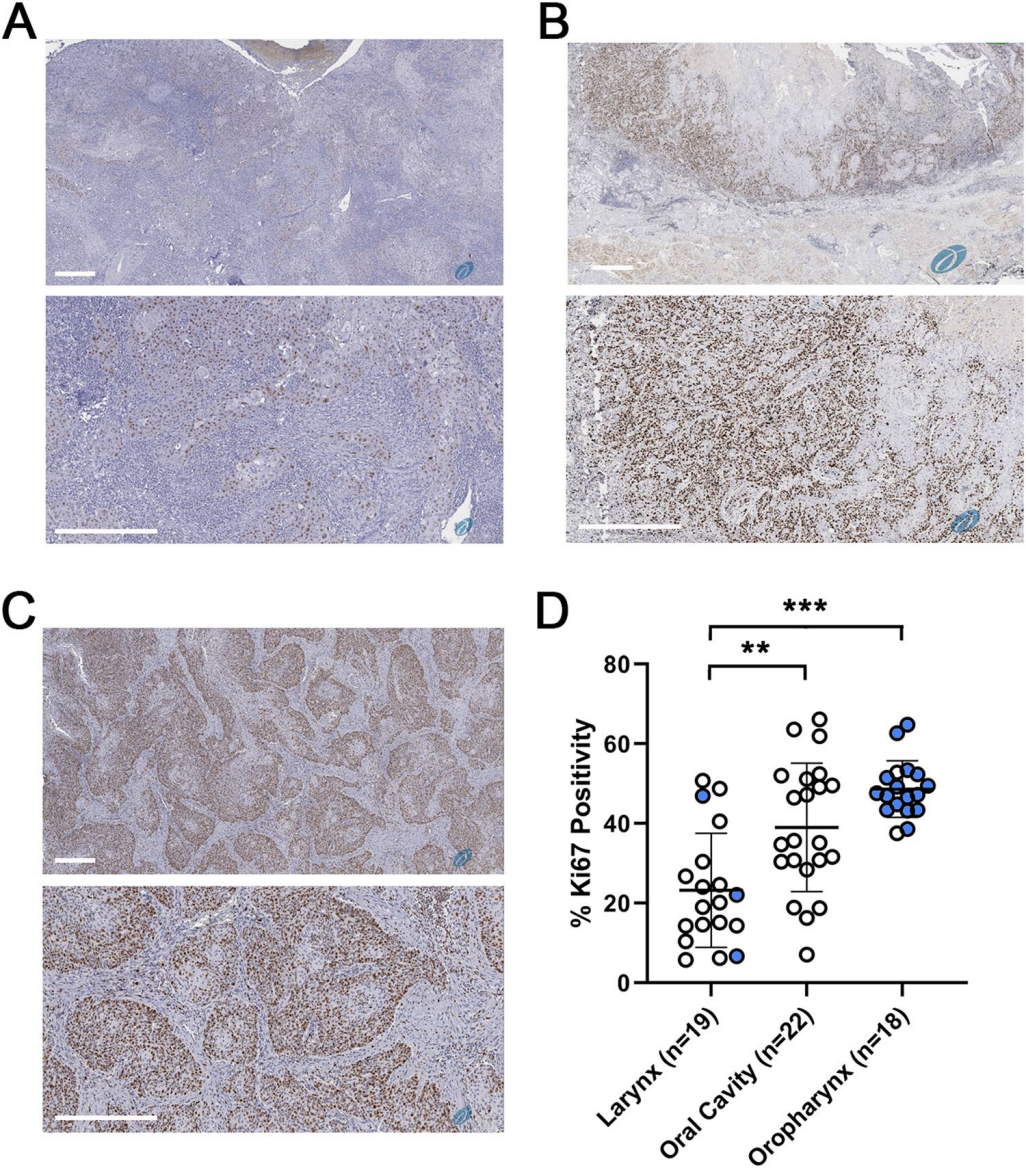
Ki-67 immuno-reactivity is increased in oral cavity SCC and oropharynx SCC compared with larynx SCC. (**A**) Representative larynx SCC section incubated with anti-Ki = 67 antibodies and visualized using the Aperio platform. (**B**) Representative oral cavity SCC section incubated with anti-Ki = 67 antibodies and visualized using the Aperio platform. (**C**) Representative oropharynx SCC section incubated with anti-Ki = 67 antibodies and visualized using the Aperio platform. Bar = 400 μM. (**D**) Graph shows % Ki-67 positive tumor cells quantified using Aperio ImageScope for 19 larynx SCC, 22 oral cavity SCC, and 18 oropharynx SCC. Bar shows mean +/− standard deviation with individual points shown as circles. Blue indicates P16 positive samples. P16 not assessed in 2 larynx SCC and 11 oral cavity SCC. **p < 0.01, ***p < 0.001.

We next compared Ki-67 immuno-reactivity as a marker of tumor cell proliferation in similarly sized SCC excised from the larynx, oral cavity, and the oropharynx, from Thomas Jefferson University Hospital from 2015–2018. Comparison of tumors 2–4 cm in size showed oral cavity and oropharyngeal tumors had significantly greater Ki67 cell positivity per tumor when compared with laryngeal tumors (Fig. 4 and Supplementary Table S1).

## Discussion

Tobacco use is the principle risk factor for developing HNSCC and therefore our findings that tobacco associated mutations are conspicuously and significantly reduced in oral cavity and oropharynx SCC compared with larynx SCC presents a number of intriguing possibilities with regards to the direct effects of tobacco smoke on somatic mutations in HNSCC. It is important to note that we are not the first to identify a difference between larynx and other sub-sites with regards to tobacco smoke mutation signatures^29,30^. However, these prior studies either used relative ratios of single base pair changes^29^, or compared COSMIC signatures not previously extracted from HNSCC datasets (https://cancer.sanger.ac.uk/signatures_v2/matrix.png) with exclusion of signature 5^30^. We show here that the increased mutation burden in larynx SCC noted by others is a direct result of tobacco smoke-associated signature 4 mutation load (Fig. 1, Supplementary Figs. S2 and S5).

The observation that larynx SCC have significantly more signature 4 mutations than other major sub-sites of HNSCC indicates that larynx SCC are more susceptible to miss-replication after DNA adduct formation leading to mutation. We speculate that the reason for this could be due to a number of possible hypotheses or combinations thereof. The first hypothesis is simply anatomical location; the larynx could be exposed to more tobacco smoke saturated mucous than other HNSCC sub-sites due to its proximity to trachea and upper lung airways. The second hypothesis is that the larynx harbor mutations in DNA repair genes which sensitizes this anatomical location to mutations from tobacco smoke. However, mutation analysis has not identified differences in somatic mutation of repair genes^11–14^ and CNA differences could well result from replication pressures associated with increased mutation burden. The third hypothesis for DNA adduct mutation susceptibility could be inherent differences in keratinocytes which line the laryngeal cavity as this structure differs from others in the oral cavity with respect to developmental origin; the larynx emerges from the foregut endoderm while much of the oral cavity emerges from the oral ectoderm^31,32^. Endoderm-derived keratinocytes may have differential DNA repair capabilities compared with ectodermal derived keratinocytes, regardless of somatic driver-gene mutations. This may also influence the number of signature 5 mutations which presumably arise through miss-replication of DNA during cell division. However, at the time of writing we were not aware of any literature addressing this possibility.

The last hypothesis is that steady state keratinocytes, regardless of anatomical location, do not differ in their ability to repair DNA adducts and the accumulation of signature 4 mutations is a factor of tumor growth over time. In this model, signature 4 mutations accumulate over time, and would predict that larynx SCC take longer to develop than those arising in the oral cavity or oropharynx.

Whilst the analysis presented in our study is descriptive in nature and we have not carried out experiments to test this model directly, there are aspects of our data and that of others which support our last hypothesis. Firstly, signature 5 mutations, associated with age in other SCC and also tobacco use, are increased in number in larynx SCC (Figs. 1 and 3) and whilst this could indicate a deficiency in DNA repair (as signature 5 mutations are associated with replication errors) the data fit with the notion of older tumors in the larynx and is supported by the observation that signature 5 mutations increase with cellular proliferation^28^. Furthermore, the proliferative index in larynx SCC was significantly less than size-matched oral cavity and oropharyngeal SCC (Fig. 4) which would support the idea that larynx SCC are slower growing and this would be a plausible explanation for increased exposure to mutagens and mutation burden. Our analysis of the age of non-smoking patients suggests that those with larynx SCC are older which could support this notion, however statistical significance was only seen compared with oropharynx SCC (Fig. 3C) and is likely confounded by HPV infection where patients are generally younger^33^. Unfortunately, the numbers of HPV positive tumors in the larynx and HPV negative tumors in oropharynx assessed in Fig. 4 are too small to determine the influence of HPV status on Ki67 proliferation index and we note conflicting results in the literature^34,35^ which together with our data advocate further interrogation of this issue.

One potential confounder to these observations that must be acknowledged is the similarity between signature 4 and signature 5. Whilst signature 5 is relatively evenly distributed (affecting all nucleotide mutation combinations relatively equally) it is the only other mutation signature found in HNSCC that has a substantial contribution of C > A nucleotide change (Supplementary Fig. S5B). Signature 4 is dominated by C > A transversions but is also similar to signature 5 in that the rest of the nucleotide variations are also relatively evenly distributed, albeit at a lower proportion than C > A. Therefore the ability to distinguish between the two, especially in tumors with limited mutation numbers is challenging. Regardless of this, it is clear that those larynx SCC positive for signature 4 harbor the most mutations compared with all other HNSCC combinations and the observation that signature 4 mutation dominates larynx SCC is well supported (Fig. 1, Supplementary Figs. S2 and S3A,B, S5).

The data presented here and our proposed model of larynx SCC developing over longer periods of time coupled with susceptibility to tobacco smoke mutations, are in keeping with a number of prior investigations and observations. Multi-region exome sequencing has shown that larynx SCC have greater heterogeneity compared with oral cavity SCC^36^ and it is conceivable that heterogeneity develops over time with an older tumor accumulating multiple clonal mutations^37^. Epidemiological observations by Doll and colleagues demonstrate that risk of death in smokers who have given up at age 30 are similar to those individuals who have never smoked, and only every year after 30 that an individual smokes increases your risk^38^. These observations would also be congruent with mutations in either the upper aero-digestive tract or lungs occurring over time, as cells age and lose DNA repair efficiency.

Given that not all smokers develop cancer (only 24% of male smokers and 11% of female smokers die from lung cancer over their lifetime^39^), a greater understanding of the factors and mechanisms that identify those smokers who have higher cancer risk could lead to prevention or early detection approaches. Further work to test our model of DNA adduct mutation susceptibility in laryngeal keratinocytes may provide insight on this subject.

In summary, our data show that larynx SCC are more susceptible to mutations associated with tobacco product DNA-adduct formation and suggest that SCC in the larynx take longer to progress compared with other HNSCC.

## Materials and Methods

### Mutation signature analysis

Our original, published analysis, used methodology from the Wellcome Trust Sanger Institute (WTSI) to extract signatures of mutation from exome sequencing of HNSCC samples^27^. Briefly, mutational signatures were extracted using 96 nonnegative components (singlebase somatic substitutions and their immediate sequence context) and compared to the validated consensus mutational signatures in COSMIC, version 2 (https://cancer.sanger.ac.uk/cosmic/signatures_v2) to identify the set of COSMIC mutational signatures in TCGA data sets^40^. This analysis identified COSMIC signatures 1, 2, 4, 5, 7, 13, and 18. Here we repeated signature assignation using the program deconstructSigs^41^ focusing on TCGA single nucleotide variation for the initial data freeze of HNSCC samples^14^ downloaded from the National Cancer Institute GDC Data Portal (https://portal.gdc.cancer.gov/projects/TCGA-HNSC). Initially we included all signatures identified using the WTSI method and comparison of deconstructSigs (using normalization within the sample and a cutoff of 0.01) with WTSI showed good concordance on results (Supplementary Fig. S6A,B) and analysis of anatomical sub-site and signature 4 and signature 5 presence were not different (Supplementary Fig. S6C,D c.f. Fig. 1B,C). A single sample from the lip showed a large contribution of signature 7 while other samples from a diverse range of non-sun exposed anatomical sub-sites showed much lower contribution of signature 7 (<40 percent in a given sample) regardless of method used (Supplementary Fig. S6B, lower panel). Because signature 7 is associated with UV exposure and not expected to be active in non-sun exposed sites, we repeated deconstructSigs analysis with exclusion of the single lip sample and signature 7. Since this study, V3 of COSMIC signatures have now been established^42^.

### Ki67 proliferation index

This retrospective study of archival, anonymized, diagnostic material was approved by the internal review board of Thomas Jefferson University and the need for written informed consent was waived by the same internal review board. All subsequent experiments were performed in accordance with relevant guidelines and regulations. Specimens of laryngeal, oral cavity and oropharyngeal SCC were retrospectively searched for in the SCC Tumor Biorepository (Sidney Kimmel Cancer Center, Thomas Jefferson University, Philadelphia, PA) from 2015 to 2018. As this analysis is comparative, we took care to select specimens that were uniform regarding tumor size as dictated by the final pathology report. We randomly selected formalin-fixed, paraffin embedded blocks of 60 cases from different patients, with enough material for new sections, compromising 19–22 specimens for each group. HPV positivity was previously determined on the basis of positive P16 immuno-histochemistry. Of the three larynx samples that were positive for P16, one was subjected to HPV *in situ* hybridization for HPV16 and HPV18 and tested positive for HPV18. Of the 16 oropharynx samples that were positive for P16, 11 were subjected to HPV *in situ* hybridization for HPV16 and HPV18, and one tested positive for HPV18, eight tested positive for HPV16, and 2 were negative. All of the selected cases were sectioned and stained for Ki67. One case was excluded from the oropharyngeal SCC group due to inappropriate immunohistochemical reaction for Ki67. At the end, the laryngeal SCC group consisted of 19 samples (n = 19), the oral cavity group consisted of 22 samples (n = 22), and the oropharyngeal SCC group consisted of 18 (n = 18). Three representative fields in each group were picked at 400x magnification (0.2 mm^2^/field) with the operator being blinded to tumor sites. All positive and negative nuclei of neoplastic cells were analyzed with the aid of Aperio ImageScope (Leica Biosystems Inc. Buffalo Grove, IL). The percentage of positive nuclei per total recorded nuclei was then noted. Supplementary Table S1 provides details of each of the samples included in Fig. 4.

### Statistical analysis

For comparison of smoking status between larynx, oral cavity and oropharynx we applied the proportional adjacent-categories-ratio model using the R package VGAM^43^. This model shows that compared to larynx, both oral cavity and oropharynx patient groups have lower probability of more severe smoking status (adjacent-categories-ratio of 0.505 and 0.491, respectively, both p < 0.001), while there is no significant difference between the oral cavity and oropharynx group. Remaining statistical analysis was performed using Prism 8 (GraphPad Software, La Jolla, CA). For comparison of mutation numbers and mutations signatures the Mann-Whitney U test was used. Pearson correlation was used to analyze relationships between age and mutation signature numbers and unpaired t-test was used to analyze Ki-67 proliferation index between separate tissue group immuno-histochemistry.

## Supplementary information

Supplementary Table S1

Supplementary Information

## Data Availability

All data are available on request.
