# Supplementary material for: Mutation signature analysis identifies increased mutation caused by tobacco smoke associated DNA adducts in larynx squamous cell carcinoma compared with oral cavity and oropharynx
Source: Sci Rep. 2019 Dec 17;9:19256. doi: 10.1038/s41598-019-55352-y (PMC6917707; doi:10.1038/s41598-019-55352-y)

Supplementary Information

Mutation signature analysis identifies increased mutation caused by tobacco smoke associated DNA adducts in larynx squamous cell carcinoma compared with oral cavity and oropharynx.

Andrew P. South, Nicoline Y. den Breems, Tony Richa, Uche Nwagu, Tingting Zhan, Shiv Poojan, Ubaldo Martinez-Outschoorn, Jennifer M. Johnson, Adam J. Luginbuhl, Joseph M. Curry.

Figure S1**:** COSMIC signature 4 contributes to a greater proportion of mutation burden in larynx compared with minor anatomical sub-sites of HNSCC. **A:** Pie charts show the proportion of mutations attributed to each of the six COSMIC mutation signatures identified in minor anatomical sub-sites of head and neck SCC. **B:** Box and whisker graphs show single nucleotide mutations attributed to signature 1, signature 2, signature 13, and signature 18, identified in each of the major subtypes of HNSCC. x = mean.

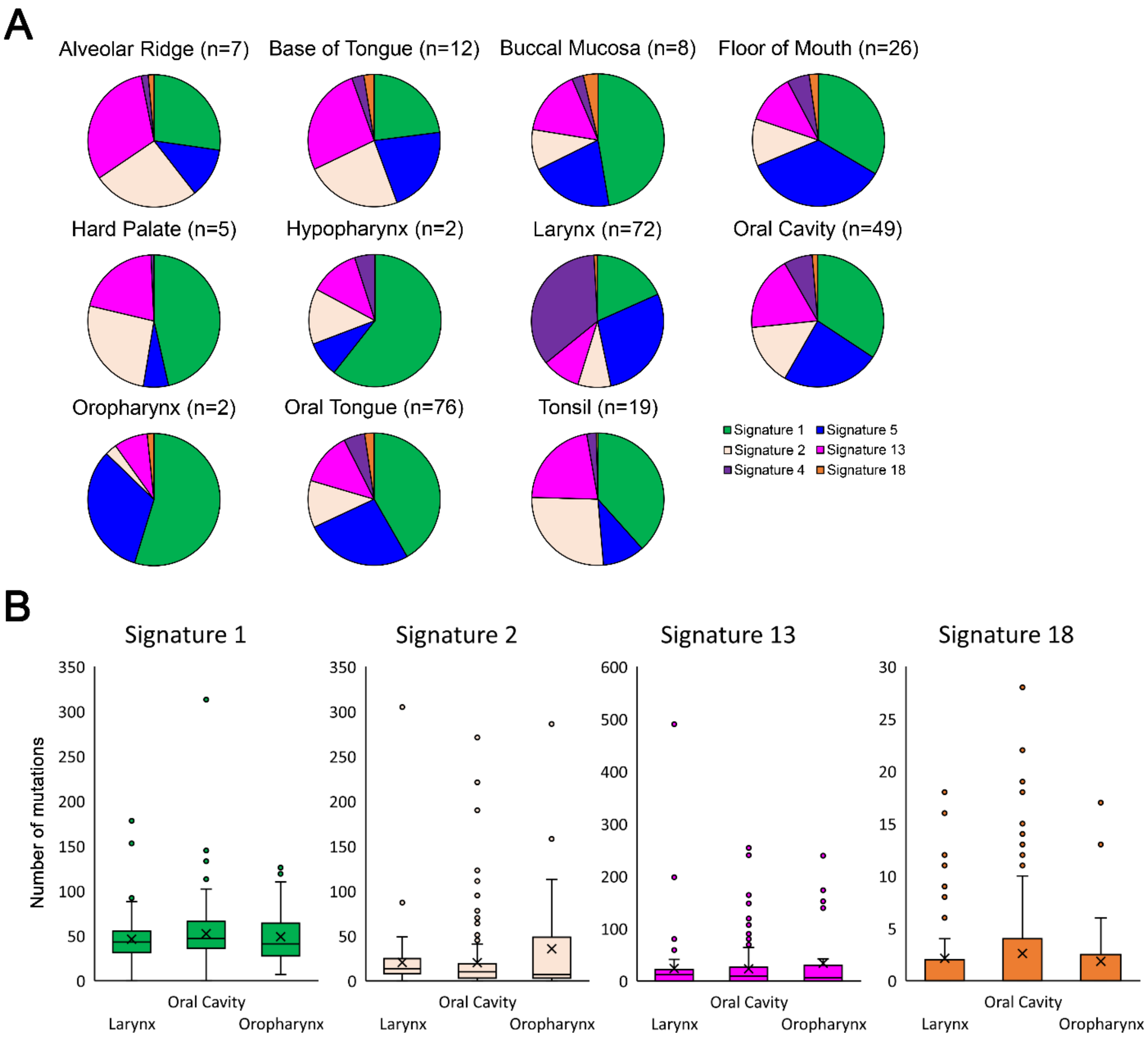

Figure S2: COSMIC signature 4 or total mutation burden is not influenced by HPV status in oral cavity and oropharynx SCC. **A**: Box and whisker graphs show total number of single nucleotide mutations as well as those attributed to COSMIC signature 4 and signature 5, identified in HNSCC stratified by presence or absence of HPV. **B**: Box and whisker graphs show total number of single nucleotide mutations as well as those attributed to signature 4 and signature 5, identified in oral cavity and oropharynx HNSCC stratified by presence (n=34) or absence (n=170) of HPV. **C**: Box and whisker graphs show total number of single nucleotide mutations as well as those attributed to signature 4 and signature 5, identified in oropharynx HNSCC stratified by presence or absence of HPV (HPV positive oropharynx n=22, HPV negative oropharynx n=11). x = mean. * = p <0.05, ** = p <0.01, *** = p<0.001.

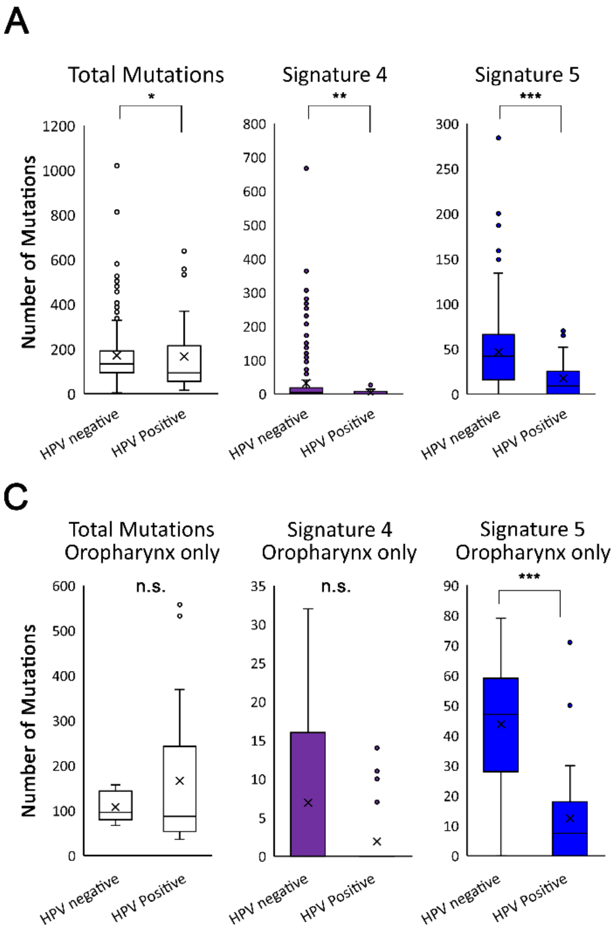

Figure S3: COSMIC signature analysis of 278 TCGA HNSCC using deconstructSigs

A: Overall mutation numbers assigned to COSMIC Signature 1, 2, 4, 5, 13, and 18, for 278 TCGA HNSCC stratified by major anatomical sub-site (larynx n=72, oral cavity n=171, oropharynx n=33, hypopharynx n=2) and ordered by mutation number (y-axis). **B**: Normalized signature contributions (signature weight, y-axis) for each tumor shown in **A**.

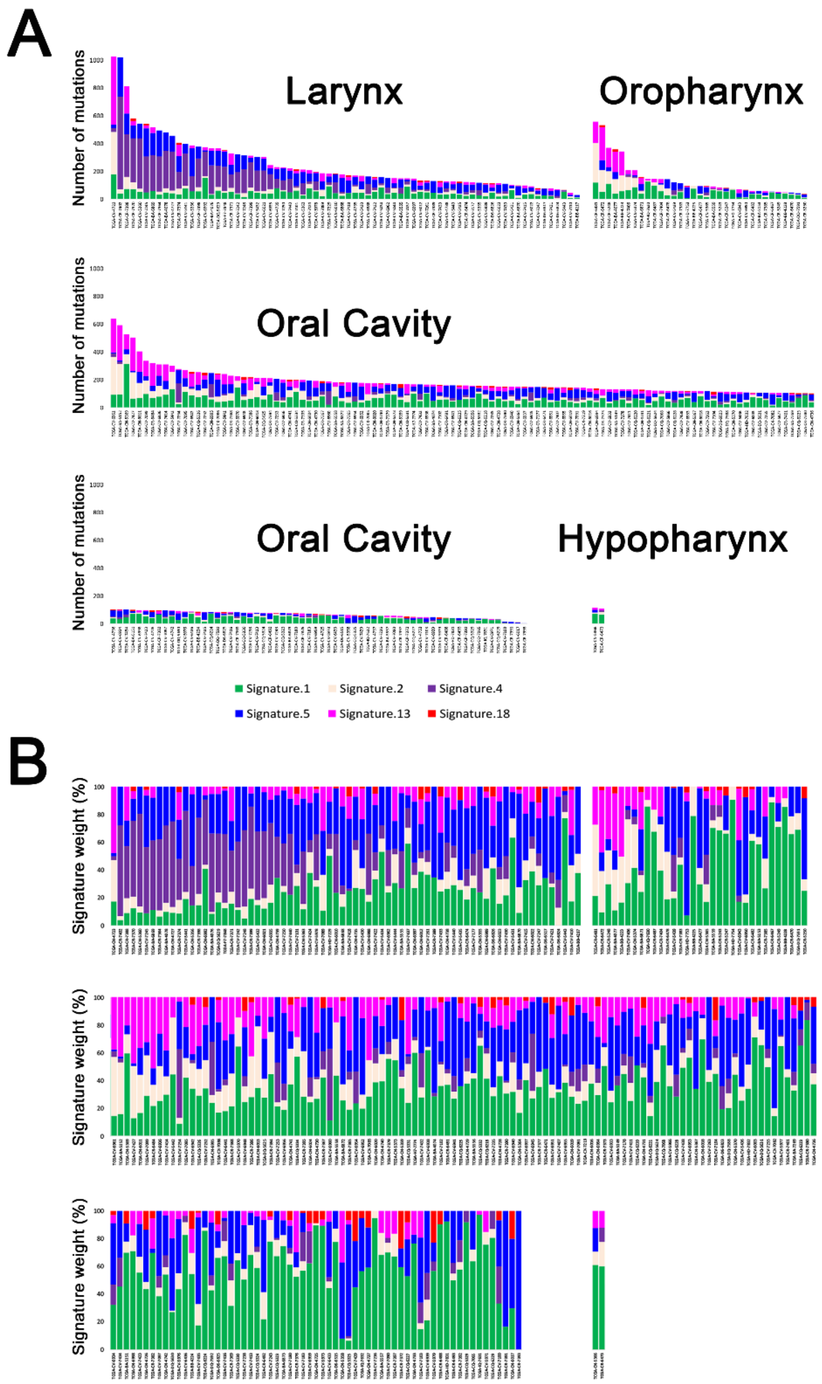

Figure S4**:** Signature 1 mutations shows positive correlation with age in all HNSCC. Graphs shows the number of signature 1 mutations (y-axis, top graph) or signature 5 mutations (y-axis, bottom graph) as determined by deconstructSigs and plotted against the age (x-axis) for all 278 TCGA HNSCC from non-sun exposed sites. These data are in line with previously published observations using the WTSI framework for mutation signature assignation[23]. Pearson Correlation r and p values given.

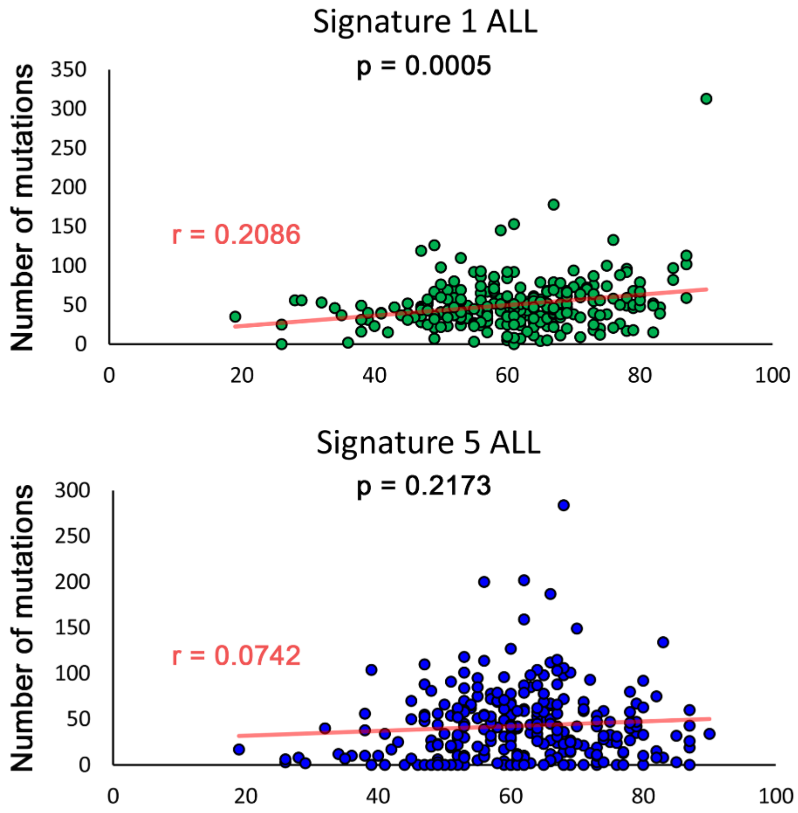

***Figure S5*: A:** 96 nucleotide context for larynx, oral cavity and oropharynx mutations are shown. X-axis depicts germline trinucleotide sequence while the corresponding color and legend above each graph depicts the nucleotide change in the tumor. Y-axis depicts the number of mutations for each of the 96-nucelotide mutations and context for a given tumor. **B:** Signature 4 and Signature 5 96 nucleotide context graphs reproduced from COSMIC Mutation Signatures version 2 (https://cancer.sanger.ac.uk/cosmic/signatures_v2).

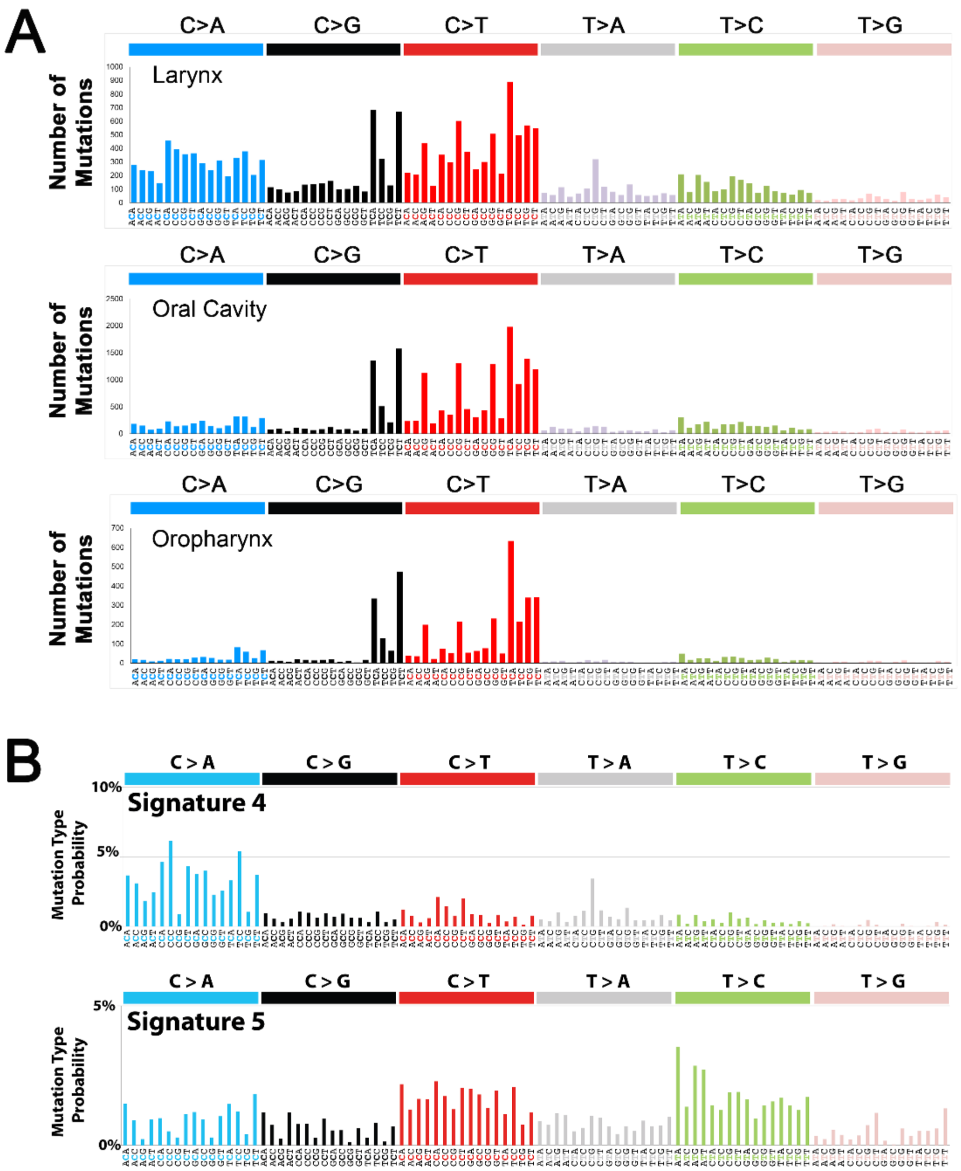

***Figure S6*: A:** Pie charts show percentage of signature assignation using either the Wellcome Trust Sanger Institute (WTSI) framework or deconstructSigs for all 279 TCGA HNSCC samples. **B:** Comparison of deconstructSig (x-axis) with WTSI signature weights. **C**: Box and whisker graphs show total number of single nucleotide mutations attributed to COSMIC mutation signatures using WTSI framework for each of the major sub-site of HNSCC. **D**: Total mutations stratified by the presence (positive) or absence (negative) of signature 4 mutations identified by the WTSI framework. x = mean. ** = $p < 0.01$, *** = $p < 0.001$.

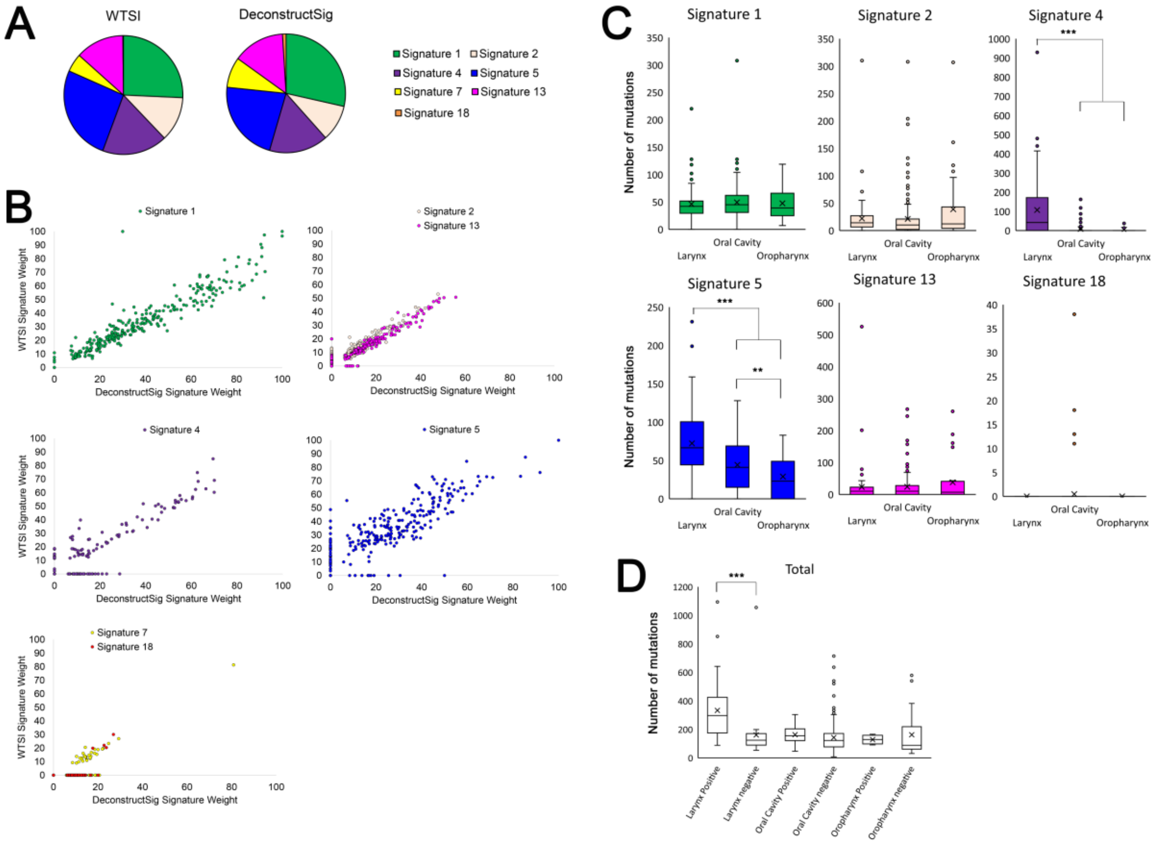

Supplement: Supplementary file 2 — Supplementary Information [file 41598_2019_55352_MOESM2_ESM.pdf]
